# Complete Genome Sequence of a *Burkholderia mallei* Isolate Originating from a Glanderous Horse from the Kingdom of Bahrain

**DOI:** 10.1128/genomeA.01296-16

**Published:** 2016-12-01

**Authors:** Mandy C. Elschner, Prasad Thomas, Falk Melzer

**Affiliations:** Friedrich-Loeffler-Institut, Federal Research Institute for Animal Health, Institute of Bacterial Infections and Zoonoses, Jena, Germany

## Abstract

*Burkholderia mallei* is a zoonotic agent causing glanders, a notifiable disease in equines. During the past decades glanders emerged, and the Kingdom of Bahrain reported outbreaks to the World Organization of Animal Health in 2010 and 2011. This paper presents the complete genome sequence of the *Burkholderia mallei* strain 11RR2811 Bahrain1.

## GENOME ANNOUNCEMENT

Glanders in horses, caused by the bacterium *Burkholderia mallei*, is a notifiable disease to the World Organization of Animal Health (OIE) and outbreaks result in massive economically relevant restrictions for trade with equids and their products for at least 6 months (1). In 2010 and 2011 Bahrain reported two events consisting of several cases in horses, most of them found in northern governorates (2). For the outbreak in 2010, infection of a dromedary was also reported (3). Genomic characterization of several isolates from this area by multilocus variable number tandem repeat analysis based on 23 different loci showed the involvement of multiple *B. mallei* strains in these outbreaks (4).

The *B. mallei* strain 11RR2811 Bahrain1 was isolated from a nasal swab collected from a glanderous horse in Bahrain 2011 and was sent to the OIE reference laboratory for glanders at the Friedrich Loeffler Institut, Federal Research Institute for Animal Health, Institute of Bacterial Infections and Zoonoses, Jena, Germany. The propagation and identification was performed following the OIE manual (5, 6). High-quality bacterial genomic DNA was extracted using the MasterPure complete DNA and RNA purification kit (Epicentre, Illumina, Madison, USA). To this end the bacterial cells were grown on nutrient agar containing 4% glycerol and harvested by rinsing with PBS solution. Nucleic acid extraction was done as per manufacturer’s recommendations.

Genome sequencing was carried out by single molecule real time (SMRT) DNA sequencing (7) using PacBio RSII sequencer generated from genomic DNA at GATC Biotech (Germany). Genome assembly was done using Hierarchical Genome Assembly Process (HGAP) algorithm version 3 (8) implemented in PacBio SMRT portal version 2.3.0. The HGAP 3 assembly generated two contigs representing the genome for the *B. mallei* strain 11RR2811 Bahrain1. The overlapping regions of circular sequences were determined using Gepard software (9). For the circularization of contigs, Circlator was used (10). The circular contigs were finally polished with RS_Resequencing.1 protocol in SMRT portal v2.3.0 and visualization was carried out in SMRT View tool (PacBio).

The final circular chromosome 1 and chromosome 2 were 3,553,991 and 2,226,175 bp with a G+C content of 68.2% and 69.0%, respectively. The annotation (NCBI Prokaryotic Genome Annotation Pipeline) identified 5,003 protein coding genes, 10 rRNA, four noncoding RNA (ncRNA), and 56 tRNA genes, in the genome.

### Accession number(s).

This whole-genome sequencing project has been deposited at DDBJ/EMBL/GenBank under the accession numbers CP017175 (chromosome_1) and CP017176 (chromosome_2). The versions described in this paper are the first versions.
